# Assessing preschoolers’ approaches to learning in the Chinese context: a scale for teacher-parent co-evaluation

**DOI:** 10.3389/fpsyg.2023.1098506

**Published:** 2023-06-02

**Authors:** Lina Feng, Jingjing Wang

**Affiliations:** College of Teacher Education, Ningbo University, Ningbo City, China

**Keywords:** approaches to learning, scale development, preschoolers, creativity, multi-group CFA

## Abstract

**Introduction:**

An effective assessment of preschoolers’ approaches to learning (ATL) requires multiple-reporter co-evaluation, such as teachers and parents. Based on extant research on children’s ATL combined with Chinese cultural background and educational policies, this study aims to develop an ATL scale suitable for Chinese teachers and parents to co-evaluate preschoolers’ ATL.

**Methods:**

Exploratory and confirmatory factor analysis (CFA) of the data collected from teachers (*n*=833) and parents (*n*=856) demonstrates the four-factor structure of the ATL: creativity, learning strategy, competence motivation, and attention/persistence, wherein creativity is a new dimension uncovered in the Chinese context.

**Results:**

Psychometric analysis demonstrates that the scale has good reliability and validity. Multi-group CFA further shows that the measurement model is robust and independent from reporter identity.

**Discussion:**

The current study contributes a novel and easy-to-use measurement instrument with 20 items for educational practitioners and for scholars who are interested in cross-cultural comparison or longitudinal development of Chinese children’s ATL.

## 1. Introduction

Taking risks and engaging with mistakes in meaningful ways is pivotal for children to gain new knowledge and skills. As a core concept in the area of early childhood education, approaches to learning (ATL) shape the way children navigate mistakes and adjust based on what they learn from the corrective feedback. Children with positive ATL will be more willing to engage in the trial-and-error practice, which generally leads them to learn faster than those with negative ATL and obtain greater academic achievement (McDermott et al., 2014). ATL is also an important factor for future success because it can improve children’s peer relationships and social interactions, but also reduce their problematic behaviors and learning difficulties (Razza et al., 2015; Peng, 2020). Overall, ATL constitutes a core area of early school readiness and is of great importance for children’s social, emotional, and cognitive development (Mcclelland et al., 2006; Hyson, 2008).

Since the concept of ATL was introduced, researchers have engaged in a series of discussions on its connotations. Early researchers believed that ‘approaches to learning’ is a general term covering a series of attitudes, habits, dispositions and styles that represent the behavioral disposition of children toward participation in learning activities (Kagan, 1995). Research points out that there are obvious differences in the concept of ATL (Serife, 2008). ATL is not a substitute for learning style. Learning style reflects stable personality characteristics (Price, 2004), while ATL has acquired plasticity and can be improved through a well-designed curriculum and teaching strategies (McDermott et al., 2016).

In recent years, researchers have posited that ATL reflects children’s enthusiasm for learning and engagement in learning (Hyson, 2008). ATL is regarded as an observable explicit behavior related to learning (McClelland et al., 2000; McDermott et al., 2001; Fantuzzo et al., 2007) or an adaptive behavior reflecting how children adapt to learning situations (Li-Grining et al., 2010). ATL describes children’s active, committed, and persistent behaviors in different learning situations (Hu et al., 2017). In prior work, ATL was defined as an umbrella concept that encompasses a series of motives and observable behaviors when children participate in learning activities. ATL has the characteristics of domain-general, acquired plasticity, and behavior externality (McClelland et al., 2000; McDermott et al., 2001; Fantuzzo et al., 2007).

Although Ministry of Education of the People’s Republic of China (2012) put forward in-depth issues regarding the factor structure of ATL earlier, there is no consensus on the factor structure of children’s ATL. The factors involved in children’s ATL may include initiative, curiosity, persistence, innovation and creativity, problem-solving, concentration, reasoning ability, and flexibility (Scott-Little et al., 2006; Peng, 2020). On the other hand, prior research suggests that the factors involved in children’s ATL include concentration, persistence, novelty and adventure, competence motivation, flexibility, learning attitude, problem-solving, and initiative (Fantuzzo et al., 2007; Bulotsky-Shearer et al., 2011; McDermott et al., 2012; Rikoon et al., 2012; Ziv, 2013). At the same time, based on research regarding China’s policy and cultural background, the factors involved in children’s ATL include persistence and concentration, initiative, imagination, creativity and invention, curiosity, learning attitude, etc. (Qian and Ding, 2010; Ministry of Education of the People’s Republic of China, 2012; Wu et al., 2016; Hu et al., 2017). Obviously, there are substantial inconsistencies and disputes in terms of the factor structure of ATL, which motivates us to examine the factor structure of ATL in the Chinese context.

In most empirical studies and early education policy documents, researchers and policymakers describe the core factors of ATL from multiple dimensions. The first is to establish the factor structure from the perspective of developmental psychology, educational psychology, and cultural psychology, and organize it in a meaningful way while being consistent with the way others organize content related to this field. The second is based on empirical research evidence. Based on the data of preschool education practice and empirical research, it emphasizes the organizational factors closely related to children’s learning outcomes (Hyson, 2008). Previous research based large samples has identified children’s competence motivation and persistence as two factors of ATL that are conceptually similar and consistent in time, which can predict children’s future academic performance (McDermott et al., 2014). The third is based on practical application. It has practical application value for the daily work of preschool education practitioners and the educational decision-making of educational policymakers. The factors of ATL should be easy to understand, memorize and describe, suitable for different learning environments, focusing on what preschool education practitioners and educational policymakers can use to operate.

There are two types of assessment instruments for ATL: one is teachers’ assessments of children’s learning behavior in various activities, and the other is the assessment of children’s classroom performance by researchers. For example, the Preschool Learning Behavior Scale (PLBS) is an instrument for teachers to measure children’s learning behavior from the following four perspectives: competence motivation, attention/persistence, strategy/flexibility, and learning attitude in the previous 2 months (McDermott et al., 2002). The classroom performance profile (CPP) enables teachers to measure children’s learning, social interaction, and creativity in the classroom (Crosby and French, 2010). The ‘children’s ATL observation and evaluation scale’ scores children’s learning behavior in-class activities on a five-point scale (Wang et al., 2010). Unfortunately, it is complex and time-consuming. Moreover, the 3- to 6-year-old children’s ATL observation and assessment scales are based on the children’s performance in an autonomous snowflake construction game (Zhao and Wang, 2018).

The instrument focuses on performing assessment in a specific situation and has many measurement aspects, which makes it overly complex. The existing assessment instruments for ATL have too many items and limited assessment scenarios (for example, building block construction game scenarios) and are suitable for a single age (for example, upperclassmen). The assessors not only have to complete many assessment items but also need to change assessment instruments for children of different ages, which makes the assessment of children’s ATL inefficient and serves to increase teachers’ workloads. Teachers also tend to ‘fill in at will,’ which is not conducive to obtaining accurate assessment results.

Scholars have called for a short and effective instrument for assessing preschoolers’ ATL (McDermott and Beitman, 1984; Diamond et al., 2013; Barbu et al., 2015). Although preschool teachers can observe children’s learning behavior in kindergarten (Ye and Guo, 2021), parents are children’s first teachers and create a family environment in which children can learn (Feng, 2020; Yue and Ren, 2021). Both teachers and parents are important educators in children’s growth, so the task of assessing children’s ATL development should fall on teachers and parents concurrently (Bodovski and Fa Rkas, 2008). In the Chinese cultural context, observations on children’s ATL are limited to those of a single reporter; for example, assessments of children’s ATL development being only conducted by teachers create an inability to obtain a comprehensive assessment. Therefore, a short and effective instrument for Chinese teachers and parents to assess children’s ATL should be developed.

To address this research gap, the current study is to develop an assessment instrument for 3 ~ 6 children that is suitable for different learning activities in both family and preschool settings. The assessment content and time needed are short, which is beneficial to obtaining an accurate assessment of the development level of children’s ATL. At the same time, this study attempts to expand the reporter role to parents; both teachers and parents should assess children’s ATL simultaneously to obtain a more comprehensive understanding of children’s ATL development.

## 2. The current study

ATL is externally manifested learning behaviors in the context of specific learning activities. Based on the real situation of children’s participation in learning activities, preschool teachers and parents can observe a series of learning-related behaviors displayed by children through their learning activities. Educators can properly understand the core factors of preschoolers’ ATL by understanding how children’s initiation, participation, and completion of learning activities, represent typical behavioral manifestations of underlying psychosocial processes including competence motivation, attention/persistence, learning strategies, and creativity.

Chinese policy related to the ATL of children aged 3–6 points out that “Children’s positive attitude and behavior disposition in the process of activities are valuable qualities necessary for perpetual learning and development. We should fully respect and protect children’s curiosity and interest in learning and help them gradually develop positive approaches to learning, such as showing initiative and being conscientious, fearless of difficulties, brave to explore, and willing to create.” The guide for evaluating the quality of early childhood care education points out that assessors should “fully respect and protect children’s curiosity and interest in the inquiry, and believe that every child is an active and capable learner.”

Based on the Chinese cultural background and preschool education policy requirements, as well as drawing upon transactional theories of development and empirical research findings of children’s ATL in the literature, we posit that children’s ATL includes four factors: competence motivation, attention/persistence, creativity, and learning strategies. The definition and examples for the four factors are shown in Table 1. In particular, creativity, in addition to the other common factors identified in the literature, should be embraced as a vital factor when measuring the ATL of Chinese children.

**Table 1:** Structure of factors composing children’s ATL.

Factor structure	Definition	Example
Competence motivation	This refers to children’s active and effective learning, which describes their willingness to participate in tasks and their determination to complete activities.	Children actively try to participate in new tasks, are willing to provide answers to discuss new activities, and show interest in activities.
Attention/persistence	This refers to children’s concentration, persistence and goal-orientation and describes children’s behavior in considering and persisting in difficult tasks.	Persistence in difficult activities, persistence in accordance with age characteristics, etc.
Creativity	This refers to the way children apply their original abilities, and describes their creativity in thinking and using materials.	The ability to explore new ideas, look at things from different angles, etc.
Learning strategy	This refers to children’s methods of accomplishing activities and solving problems.	Including flexibility in the process of doing things, receiving necessary help, etc.

The purpose of this study is thus to develop and evaluate the psychometric properties of the ATL, a 20-item instrument measuring ATL in early childhood education based on Ministry of Education of the People’s Republic of China (2012). First, we determined four factors of the ATL instrument across teachers and parents using scree plot and expert evaluations. Second, we ensured that the factor solution applied in both groups (i.e., teachers and parents) and, thus, we validated the ATL instrument’s structure using the multi-group CFA techniques. Finally, we examined the reliability of the ATL instrument by assessing the convergent validity and calculating its composite reliability.

## 3. Method

**Figure 1:**
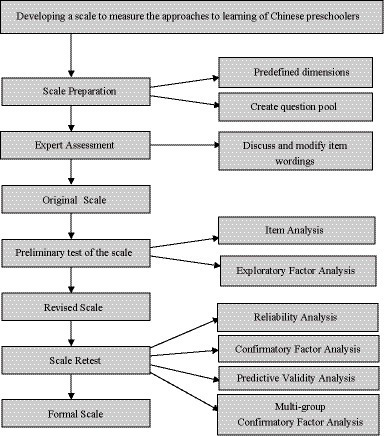
Scale development procedure of this study.

The procedure behind the ATL scale development process in this study is presented in Figure 1.

### 3.1. Participants

In this study, preschools of different regions were selected in province Z of eastern China as the sample source. Stratification sampling method was adopted to ensure the representativeness of the collected samples. We first selected preschools in different districts, then we selected classes in the preschools selected in the previous stage, and finally children were selected from the classes above. All selections keep random in the whole sampling process under the great assistance of the local educational government. Questionnaires were collected from teachers and parents of children attending these preschools. Questionnaires were distributed with the consent of both teachers and parents. Teachers and parents completed scales evaluating each child’s ATL, social skills, and executive function. Demographic information about teachers and parents was also collected, such as age, teaching standing, highest education level, annual income level, and occupation. To obtain an exploratory sample to explore the factor structure of ATL, we distributed 400 questionnaires to teachers and parents. After screening out invalid participants who provided regular responses to questionnaire items and questionnaires with missing values, we obtained 363 teacher samples and 375 parent samples.

After 2 months, we distributed 500 questionnaires to teachers and parents to collect a confirmatory sample, and finally, we obtained 470 teacher samples and 481 parent samples. The total number of teacher-version samples was 833, composed of 425 boys and 408 girls, with 28.3% in the lower class, 35.8% in the middle class and 35.9% in the upper class. The total number of parent-version samples was 856, composed of 424 boys and 432 girls. The lower class accounted for 28.2%, the middle class accounted for 35.5%, and the upper class accounted for 36.3%. The initial test samples were used for item analysis and exploratory factor analysis, and the retest samples were used for confirmatory factor analysis (CFA) and reliability and validity analysis.

### 3.2. Measures

The children’s ATL scale is composed of teachers’ and parents’ scales. There are similarities between the two versions of scales for the assessment of children’s ATL, and both include the development rating of target children in the four dimensions of competence motivation, attention/persistence, creativity, and learning strategy. There are 43 and 39 items in the teacher and parent versions, respectively. By referring to the prior work on ATL in the literature (McDermott et al., 2002, 2012; Rikoon et al., 2012), all items are rated on a 3-point Likert scale (Most often applies, Sometimes applies, or Does not apply) depicting the presence of the specific behavior during the past 2 months. It is a relatively cost-effective and unobtrusive assessment tool. The valence (positive or negative) of item wording is varied as an approach to detect invalid responses.

First, the original scale was evaluated by experts. The experts advised as to which topics are not conducive to evaluation and should be modified or deleted. The test stage was divided into the initial test and the retest. Item analysis and exploratory factor analysis were carried out on the initial test sample data. According to the analysis results, the items were deleted or modified to form the initial scale. Then, the second set of sample data was tested by CFA and reliability and validity tests. Finally, the teacher version and the parent version of the children’s approaches to learning assessment scale were formed. Each scale included the four dimensions of competence motivation, attention/persistence, creativity, and learning strategies, a total of 20 items including both positive and negative expression items. The scores of negative expression items were reversed in the data analysis. Therefore, higher scores on the overall scale suggest that children’s ATL are better.

In this study, the social skills rating scale for children (Gresham et al., 2011) was adopted to measure children’s social skills. There are 39 items in the parent version and 30 questions in the teacher version of the scale, which both include the four dimensions of cooperation, advocacy, responsibility, and self-control. The higher the total score is, the better the children’s social skill level. In addition, the executive function scale was used to assess children’s executive function (Thorell and Nyberg, 2008). The three dimensions assessed by this scale are working memory, regulation ability and inhibition ability. Five-point scoring (totally incorrect–completely correct) was used. A high score on the scale indicates a worse executive function in children (Wei et al., 2018).

### 3.3. Analytic approach

SPSS 26.0 and Mplus 7.4 were used to examine the psychometric properties of the ATL scale. First, item analysis and exploratory factor analysis were carried out based on the exploratory sample of the ATL assessment scale using SPSS 26.0. Then, the reliability and validity performance of the scale were tested using a confirmatory sample. The internal consistency coefficient and composite reliability were calculated to examine the reliability of the scale. Structural validity was tested using CFA, and predictive validity was tested by path analysis in Mplus 7.4. Finally, the structural stability of assessment by cross-rating (teacher and parent) was tested using multigroup CFA.

## 4. Results

Initially, there were 43 items in the teacher version and 39 items in the parent version. We then invited 15 experts in the field of early childhood development to evaluate the language expression and content appropriateness of the items. In accordance with several expert assessments and suggestions, we modified the scale items. Specifically, 12 experts stated that the descriptions of items TCM10, PCM10, TCR24, PCR24, TCR29, and PCR29 were not clear enough, which was not conducive to evaluation. Eight experts stated that the expressions in TAP12, PAP12, TAP15, PAP15, TAP18, and PAP18 reflect age characteristics of children’s psychological development or behavior problems, which was not suitable for the assessment of children’s ATL. After deleting TCM10, PCM10, TAP12, PAP12, TAP15, PAP15, TAP18, PAP18, TCR24, PCR24, TCR29, and PCR29, we finalized the original scale for data collection and psychometric analysis. After expert assessment, 37 items remained in the teacher version, and 33 items remained in the parent version. These items were then analyzed.

### 4.1. Item analysis

In item analysis, items are selected according to the following three criteria: critical ratio, item-total score correlation, and commonality. In the critical ratio method, a t value of less than 3 indicates that the discrimination of an item is poor and should be deleted. Items with item-total score correlation coefficients of less than 0.4 should also be removed. Moreover, the cut-off value of commonality is generally set as 0.4. A commonality value lower than 0.4 indicates that items are not closely related to common factors and should also be deleted.

#### 4.1.1. Teacher version (*N* = 363)

As shown in Table 2, TCM1, TCM2, TCM3, TCM8, TAP13, TCR19, TCR20, TCR21, TLS34, TLS36, TLS37, TLS38, TLS39, TLS40, TLS41, TLS42, and TLS43 should be deleted according to the screening criteria detailed above. In summary, there are 20 items left in the scale after applying the critical ratio method and commonality test during item analysis.

**Table 2:** Item analysis results (teacher version).

Items	*t*	*r*	Commonality	Items	*t*	*r*	Commonality
TCM1	**2.750**	0.140**	0.720	TCR25	13.961	0.601**	0.694
TCM2	3.227	0.235**	0.718	TCR26	14.396	0.607**	0.626
TCM3	**2.526**	0.152**	0.678	TCR27	13.829	0.605**	0.716
TCM4	14.907	0.590**	0.688	TCR28	13.014	0.585**	0.690
TCM5	13.220	0.589**	0.754	TLS30	19.661	0.669**	0.760
TCM6	13.357	0.578**	0.711	TLS31	16.424	0.624**	0.686
TCM7	13.546	0.591**	0.744	TLS32	14.738	0.604**	0.736
TCM8	3.340	0.234**	0.602	TLS33	15.308	0.586**	0.723
TCM9	13.645	0.596**	0.681	TLS34	**1.869**	0.114**	0.538
TAP11	12.417	0.550**	0.690	TLS35	14.846	0.598**	0.688
TAP13	**1.275**	**0.093**	0.473	TLS36	**1.940**	0.104*	0.724
TAP14	12.574	0.557**	0.674	TLS37	4.177	0.216**	0.731
TAP16	11.692	0.541**	0.693	TLS38	**2.734**	0.128*	0.746
TAP17	14.142	0.586**	0.685	TLS39	**1.776**	0.127*	0.714
TCR19	4.683	0.262**	0.807	TLS40	**−0.512**	**−0.016**	0.579
TCR20	4.514	0.277**	0.738	TLS41	3.439	0.243**	0.705
TCR21	**2.744**	0.145**	0.561	TLS42	**0.660**	**0.073**	0.725
TCR22	14.026	0.590**	0.650	TLS43	**0.340**	**0.053**	0.738
TCR23	14.446	0.588**	0.682				

*p < 0.05, **p < 0.01. The bold values indicate the *t* < 3 and *r* < 0.4.

#### 4.1.2. Parent version (*N* = 375)

As shown in Table 3, after using the screening criteria in the item analysis, PCM1, PCM2, PCM3, PCM8, PAP13, PCR19, PCR20, PCR21, PLS34, PLS36, PLS37, and PLS39 should be deleted. In summary, there are 21 questions left in the children’s approaches to the learning assessment scale (parent version) after the combination of the critical ratio method, item-total score correlation and commonality test during item analysis.

**Table 3:** Item analysis results (parent version).

Items	*t*	*r*	Commonality	Items	*t*	*r*	Commonality
PCM1	4.215	0.365**	0.656	PCR23	12.346	0.545**	0.600
PCM2	**0.204**	**0.025**	0.634	PCR25	14.108	0.564**	0.609
PCM3	3.163	0.193**	0.312	PCR26	13.004	0.563**	0.587
PCM4	12.050	0.513**	0.644	PCR27	15.232	0.588**	0.605
PCM5	15.323	0.590**	0.661	PCR28	14.902	0.580**	0.641
PCM6	12.494	0.548**	0.612	PLS30	13.406	0.573**	0.648
PCM7	12.025	0.538**	0.576	PLS31	13.159	0.560**	0.667
PCM8	**2.879**	0.253**	0.473	PLS32	14.285	0.590**	0.636
PCM9	12.072	0.545**	0.610	PLS33	15.261	0.601**	0.668
PAP11	16.402	0.612**	0.771	PLS34	**1.124**	**0.051**	0.545
PAP13	**0.336**	**0.077**	0.635	PLS35	13.917	0.577**	0.652
PAP14	14.877	0.597**	0.711	PLS36	**0.104**	0.040	0.626
PAP16	15.532	0.614**	0.757	PLS37	**0.746**	0.102*	0.588
PAP17	12.963	0.565**	0.741	PLS38	13.326	0.690**	0.619
PCR19	**2.098**	**0.088**	0.485	PLS39	**1.140**	0.125*	0.463
PCR20	3.134	0.205**	0.643				
PCR21	3.757	0.280**	0.475				
PCR22	13.491	0.573**	0.599				

*p < 0.05, **p < 0.01. The bold values indicate the *t* < 3 and *r* < 0.4.

### 4.2. Exploratory factor analysis (teacher version)

In this study, exploratory factor analysis was conducted on the 20 items of the scale that remained after item analysis to analyze the scale’s factor structure. Specifically, the principal component method (PCA) was used for factor extraction, and the varimax method was used for factor rotation. The number of factors was determined by combining eigenvalues and scree plots. KMO = 0.935, and the Bartlett spherical test reached a significant level (*p* < 0.001), indicating that the data were suitable for exploratory factor analysis (Kaiser and Rice, 1974; Spicer, 2005). The results showed that four factors with eigenvalues greater than 1 were extracted using the principal component method (see Supplementary Table A1 for the detailed results of eigenvalues and explained variance proportion). The cumulative explanatory variation in the four factors reached 69.115%, which was more than the commonly used threshold of 50% in the field of education psychology, indicating that the extracted factors can account for most of the total variance in items (Sparkman et al., 1979). The scree plot also suggests that the ATL may have four factors (Sun and Zhou, 2005; see Supplementary Figure A1). Combining the scree plot and the factor eigenvalues, four factors were chosen as the appropriate number for the teacher version of the ATL scale.

Table 4 shows the factor loadings of items after factor rotation. There are six items in factor 1, which are all related to children’s creativity. Therefore, the factor is named “creativity.” Factor 2 has five items related to children’s learning strategies. There are five items in factor 3, which are all related to children’s competence motivation. Therefore, the factor is named “competence motivation.” Factor 4 has four items, which are related to children’s attention or persistence. Therefore, the factor is named “attention/persistence.” In general, the number of factors and item-factor correspondences obtained by exploratory factor analysis are consistent with our theoretical expectation.

**Table 4:** Factor loading matrix after rotation (teacher version).

Items	Factor			
	1	2	3	4
4. Hesitant to talk about new activities	0.199	0.163	**0.772**	0.143
5. Thinks tasks are too hard, makes no attempt	0.094	0.235	**0.808**	0.143
6. Easily gives up on activities	0.150	0.182	**0.790**	0.158
7. Depends on adults for direction	0.185	0.219	**0.800**	0.123
9. Lively interest in activities	0.269	0.095	**0.759**	0.170
11. Cooperates in group activities	0.263	0.100	0.156	**0.763**
14. Sticks to age appropriate activities	0.121	0.246	0.191	**0.752**
16. Pays attention to what the teacher says	0.156	0.195	0.121	**0.787**
17. Insufficient time spent analyzing problems	0.189	0.224	0.191	**0.752**
22. Is aware of problems others often do not see	**0.760**	0.175	0.162	0.063
23. Imaginative in play	**0.774**	0.152	0.192	0.130
25. Willingly participates in unfamiliar group activities	**0.775**	0.110	0.198	0.179
26. Acts positive and confident in new tasks or activities	**0.723**	0.184	0.136	0.187
27. Maintains positive attitude toward new activities	**0.785**	0.173	0.167	0.118
28. Given a choice, takes on a new task rather than a familiar one	**0.767**	0.154	0.093	0.209
30. Compares new with old tasks as per what worked	0.208	**0.791**	0.227	0.187
31. Develops plan after considering possible consequences	0.225	**0.762**	0.154	0.146
32. Shows a basic understanding of cause and effect	0.171	**0.809**	0.201	0.110
33. Verbalizes possible consequences of actions or events	0.147	**0.797**	0.144	0.198
35. Communicates that problems have more than one solution	0.153	**0.755**	0.188	0.230

The bold values indicate the items with factor loading bigger than 0.5.

### 4.3. Exploratory factor analysis (parent version)

In this study, exploratory factor analysis was conducted on the remaining 21 items after item analysis to analyze the factor structure of the scale. The results showed that KMO = 0.920, and Bartlett’s spherical test reached a significant level (*p* < 0.001), indicating that there are considerable associations among items, thus meeting the premise of exploratory factor analysis. The results demonstrated that the cumulative explanatory variation in the four factors reached 63.482% (see Supplementary Table A2 for the detailed results of eigenvalues and explained variance proportion), which further indicates that the 4-factor structure has good explanatory power. The scree plot also showed that there are four factors before the inflection point, thus the 4-factor structure may be a suitable solution (see Supplementary Figure A2).

Table 5 shows that after the first factor rotation, the factor loading of item 38 “adopts specific and inflexible procedures” did not reach 0.50, and the item-factor attribution relationship was not clear. Therefore, exploratory factor analysis was conducted again after deleting item 38. The second factor rotation results showed that the 4-factor model was supported. At that time, the item-factor attribution relationship of all items became clear. Specifically, factor 1 has six items related to children’s creativity. Therefore, the factor is named “creativity.” Factor 2 has five items related to children’s learning strategies. There are five items in factor 3. The expressions of these items are all related to children’s competence motivation; thus, this factor is named “competence motivation.” Finally, factor 4 has 4 items related to children’s attention/persistence. Generally, the number of factors obtained by exploratory factor analysis is consistent with our theoretical expectation.

**Table 5:** Factor loading matrix after rotation (parent version).

Items	First-time EFA				Second-time EFA			
	F1	F2	F3	F4	F1	F2	F3	F4
4. Hesitant to talk about new activities		**0.762**		.			**0.765**	
5. Thinks tasks are too hard, makes no attempt		**0.759**		.			**0.757**	
6. Easily gives up on activities		**0.740**					**0.741**	
7. Depends on adults for direction		**0.725**					**0.732**	
9. Lively interest in activities		**0.725**					**0.719**	
11. Cooperates in group activities				**0.811**				**0.813**
14. Sticks to age-appropriate activities				**0.784**				**0.785**
16. Pays attention to what parents say	.			**0.823**				**0.824**
17. Insufficient time spent analyzing problems				**0.822**				**0.823**
22. Is aware of problems others often do not see	**0.742**				**0.745**			
23. Imaginative in play	**0.744**				**0.746**			
25. Willingly participates in unfamiliar group activities	**0.732**				**0.734**			
26. Acts positive and confident in new tasks or activities	**0.730**				**0.732**			
27. Maintains positive attitude toward new activities	**0.747**				**0.748**			
28. Given a choice, takes on a new task rather than a familiar one	**0.774**				**0.772**			
30. Manages transitions (e.g., When it is time for a story, child puts away the blocks and goes to hear the story)			**0.747**			**0.749**		
31. Adjusts behavior to correspond to different settings (e.g., child knows when to use a ‘quiet voice’)			**0.776**			**0.777**		
32. Makes independent decisions (e.g., instead of playing with friends, the child decides to read a story)		.	**0.732**			**0.733**		
33. Copes with frustration (e.g., Child says ‘We have to go inside, it’s raining. We can come back out when it stops.’)	.		**0.770**			**0.771**		
35. Follows household rules			**0.750**			**0.752**		
38. Adopts specific/inflexible procedures	0.332	0.469	0.278	0.309				

The bold values indicate the items with factor loading bigger than 0.5.

Next, two new samples for the teacher version (*N* = 470) and parent version (*N* = 481) were used to further verify the factor structure, reliability and validity of the scale.

### 4.4. Reliability analysis

Cronbach’s α and the composite reliability coefficient were used to test the reliability of the scale. To develop measurement instruments, the reliability coefficients should be above 0.70 (Strauss and Smith, 2009). As shown in Table 6, the reliability of the scale is acceptable.

**Table 6:** Reliability of ATL scale.

		Teacher version		Parent version	
Dimension	Items	Cronbach’s α	CR	Cronbach’s α	CR
Competence Motivation	5	0.875	0.920	0.854	0.873
Attention/persistence	4	0.850	0.902	0.839	0.870
Creativity	6	0.892	0.923	0.864	0.895
Learning strategy	5	0.872	0.914	0.838	0.880
Total scale	20	0.915	---	0.889	---

### 4.5. Validity analysis

#### 4.5.1. Content validity

In this study, the formulation of children’s ATL scale is based on a large number of positivist studies, education policies and related literature regarding the concept of ATL. Experts were invited to evaluate the items, and the scale was modified according to experts’ suggestions, which ensures that the scale has good content validity.

#### 4.5.2. Structural validity (teacher version)

**Figure 2:**
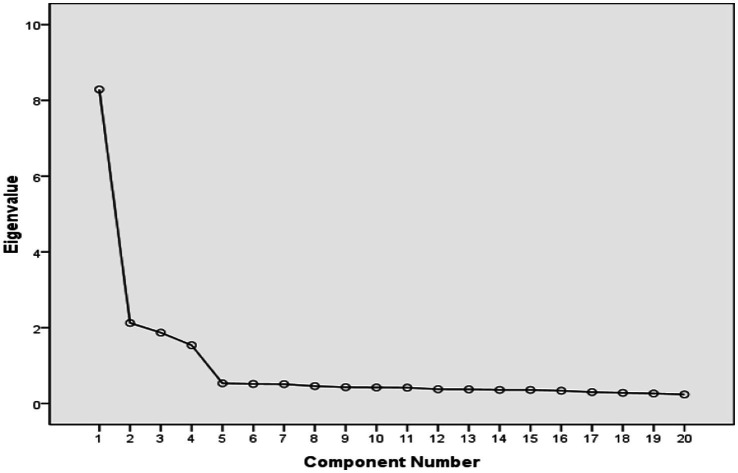
Diagram of CFA (teacher version).

The results of CFA showed that *χ*^2^ = 359.361, df = 164, *χ*^2^/df = 2.191<3, CFI = 0.958>0.900, TLI = 0.952>0.900, and RMSEA = 0.050<0.080, indicating that the model fit the data well (Hu and Bentler, 1999). Figure 2 shows that the standardized factor loadings of all items are above 0.6, and there is a medium degree of correlation among the factors. These results indicate that the structural validity of the measurement model is good.

#### 4.5.3. Structural validity (parent version)

**Figure 3:**
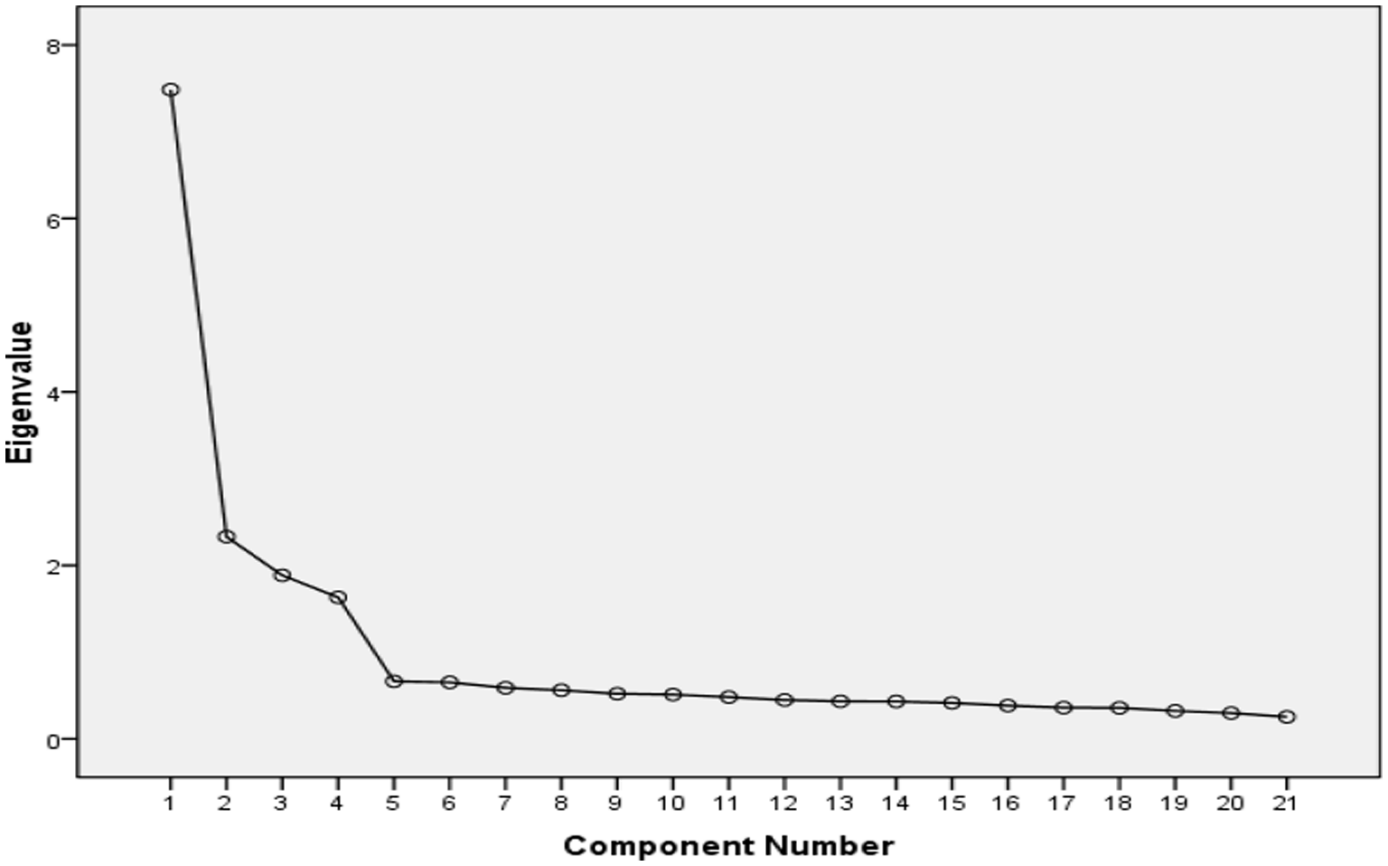
Diagram of CFA (parent version).

The results of CFA showed that *χ*^2^ = 239.107, df = 164, *χ*^2^/df = 1.458 < 3, CFI = 0.975 > 0.900, TLI = 0.971 > 0.900, and RMSEA = 0.031 < 0.050, indicating that the model fit of this factor model is acceptable. Figure 3 illustrates that the standardized factor loadings of all items are above 0.6, and there is a medium degree of correlation among the factors. These results indicate that the structural validity of the model is good.

We also examined the convergent and discriminant validity of the ATL scale. Convergent validity was assessed by inspecting the factor loadings and average variance extracted (AVE) values. The results showed that the values of AVE range from 0.666 to 0.697 for the teacher-version, and range from 0.580 to 0.626 for the parent-version (see Supplementary Table A3), both higher than the cut-off of 0.50 (Fornell and Larcker, 1981). In addition, Figures 2, 3 demonstrate that the values of all the factor loadings of scale items were greater than the recommended threshold of 0.60 (Carmines and Zeller, 1979). These results suggest good convergent validity of the dimensions in the ATL scale. Furthermore, Table 7 shows that the square roots of AVEs for all dimensions in teacher-version and parent-version scale were greater than the correlation coefficients among the dimensions, indicating good discriminant validity of the ATL measurement model in this study (MacKenzie et al., 2011).

**Table 7:** Discriminant validity of ATL scale.

Dimension	Teacher version				Parent version			
	1	2	3	4	1	2	3	4
Competence motivation	**0.835**				**0.762**			
Attention/persistence	0.555	**0.834**			0.517	**0.791**		
Creativity	0.651	0.598	**0.816**		0.443	0.540	**0.766**	
Learning strategy	0.587	0.560	0.567	**0.825**	0.493	0.480	0.489	**0.772**

Diagonal elements are the square roots of AVE.

#### 4.5.4. Predictive validity (teacher version)

**Figure 4:**
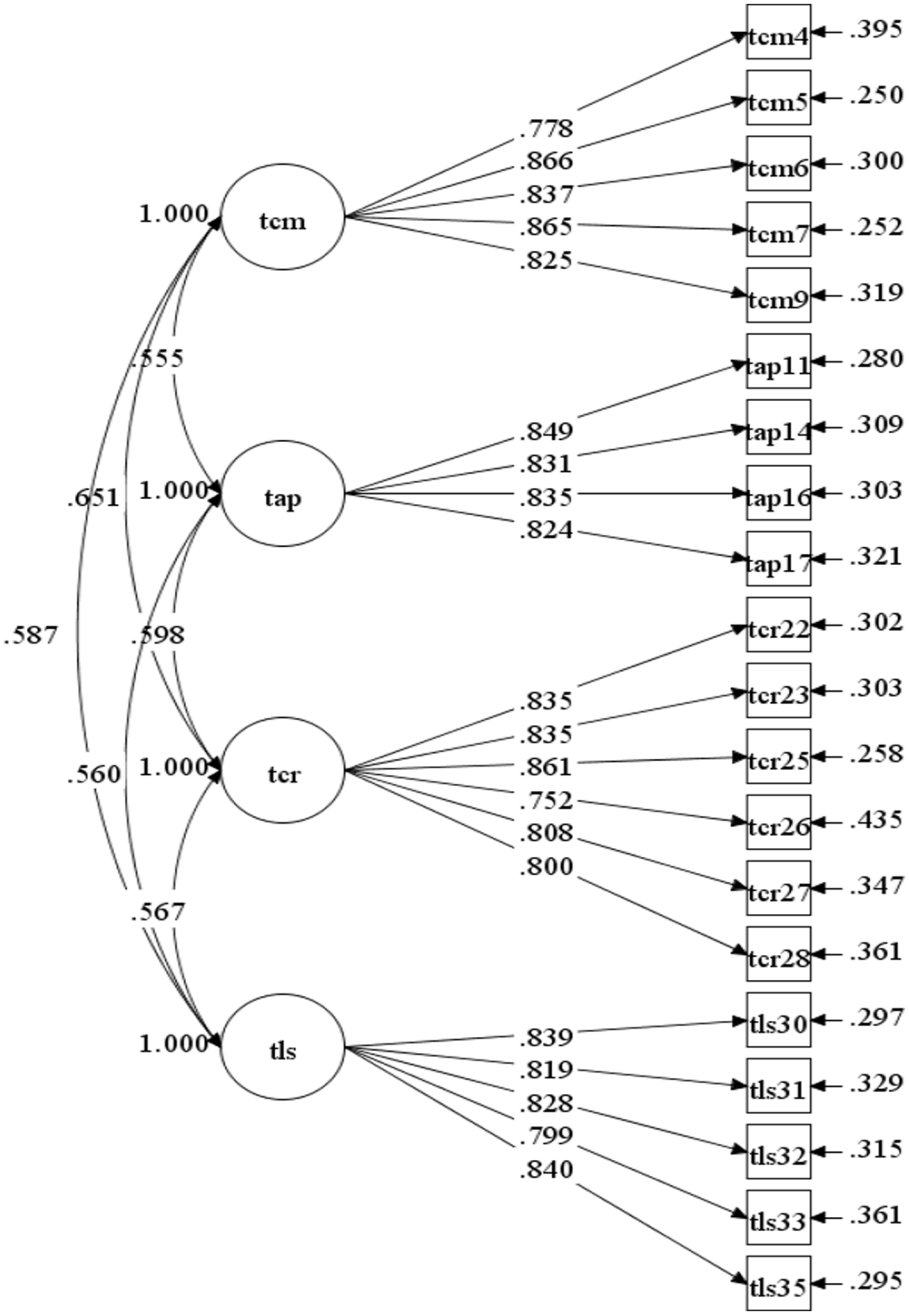
Predictive validity model (teacher version).

The results showed that *χ*^2^ = 349.442, df = 284, *χ*^2^/df = 1.230<3, CFI = 0.990>0.900, TLI = 0.989>0.900, and RMSEA = 0.022<0.050, indicating that the predictive model fits the data well (see Figure 4). As shown in Table 8, the results showed that each dimension of ATL can significantly positively predict children’s social skills and negatively predict children’s executive function. Thus, the predictive validity of the ATL scale for children (teacher version) is good.

**Table 8:** Predictive coefficients of ATL on social skills and executive function (teacher version).

Prediction path	Standardized coefficient	SE	*P*
Competence Motivation→ Social Skills	0.144	0.052	0.005
Attention/Persistence →Social Skills	0.285	0.049	***
Creativity →Social Skills	0.270	0.051	***
Learning Strategy →Social Skills	0.328	0.049	***
Competence Motivation→ Executive Function	−0.274	0.047	***
Attention/Persistence → Executive Function	−0.279	0.045	***
Creativity → Executive Function	−0.281	0.047	***
Learning Strategy → Executive Function	−0.187	0.045	***

***p < 0.001.

#### 4.5.5. Predictive validity (parent version)

**Figure 5:**
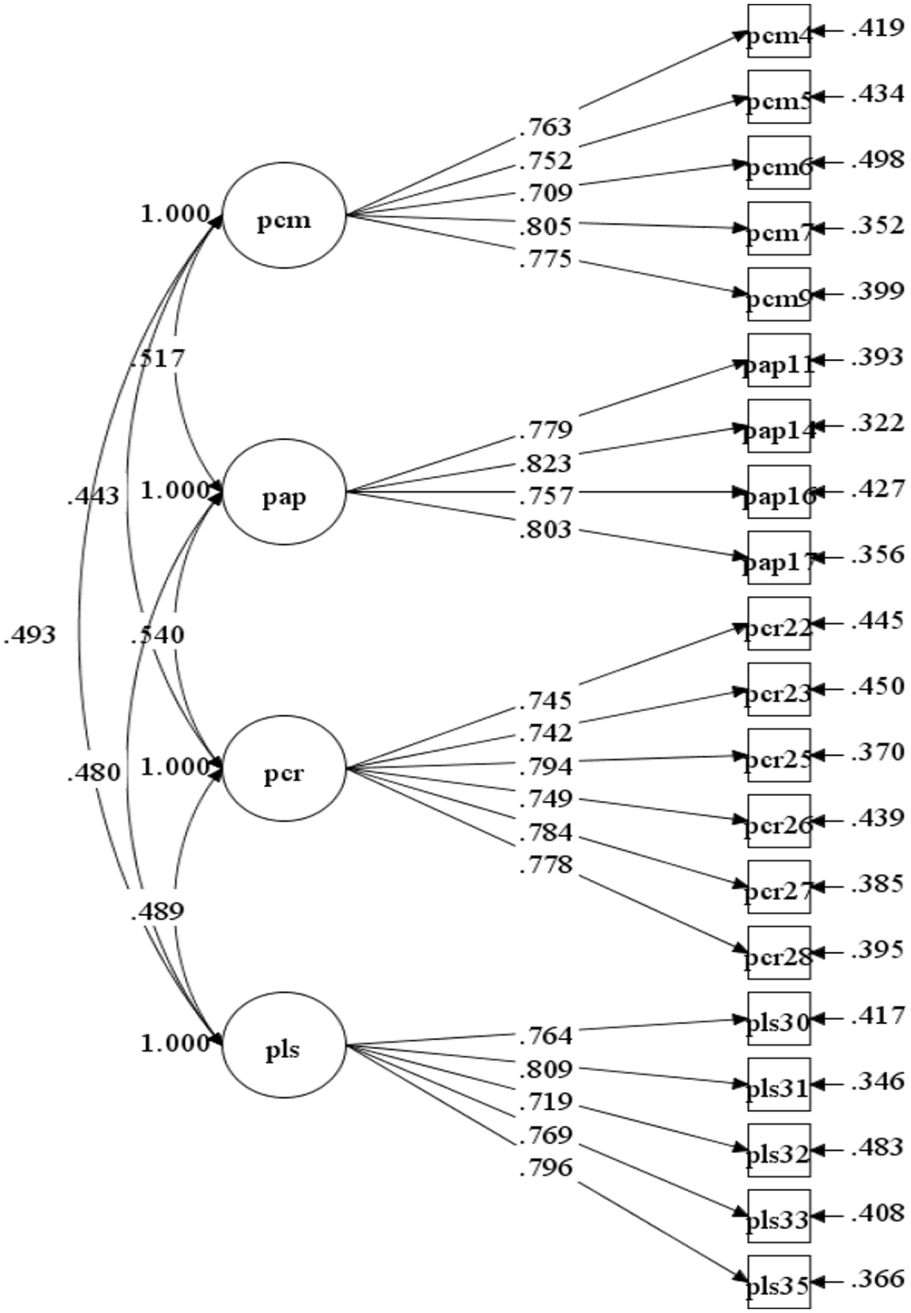
Predictive validity model (parent version).

The results show that *χ*^2^ = 341.762, df = 309, *χ*^2^/df = 1.106<3, CFI = 0.995>0.900, TLI = 0.994>0.900, RMSEA = 0.015<0.050, indicating that the predictive model fits the data well (see Figure 5). As shown in Table 9, the results showed that each dimension of ATL can significantly positively predict children’s social skills and negatively predict children’s executive function. Therefore, the predictive validity of children’s approaches to the learning scale (parent version) is acceptable.

**Table 9:** Prediction coefficients of ATL on social skills and executive function (parent version).

Prediction path	Standardized coefficient	SE	P
Competence Motivation→ Social Skills	0.173	0.044	***
Attention/Persistence →Social Skills	0.335	0.043	***
Creativity →Social Skills	0.292	0.043	***
Learning strategy →Social Skills	0.277	0.043	***
Competence Motivation →Executive Function	−0.197	0.048	***
Attention/Persistence → Executive function	−0.345	0.046	***
Creativity → Executive Function	−0.263	0.047	***
Learning Strategy → Executive Function	−0.206	0.047	***

***p < 0.001.

### 4.6. Multi-group confirmatory factor analysis

The structural equivalence between the teacher and parent versions was assessed by multi-group CFA (*N* = 470 + 481 = 951). When setting up the multiple group model, we assured that the measurement parameters of the teacher model and the parent model were freely estimated but limited the model configuration of the two groups for consistency (i.e., identical factors and item-factor relationship). The results of multi-group CFA showed that: *χ*^2^ = 585.074, df = 328, *χ*^2^/df = 1.784<3, CFI = 0.964>0.900, TLI = 0.958>0.900, and RMSEA = 0.041<0.050, indicating that the model fitting of the multi-group CFA model is acceptable. Supplementary Figures A3, A4 display the estimated results of the multi-group CFA parameters of the measurement model for teacher-version and parent-version approaches to the learning scale, respectively. Therefore, the measurement model of the scale is robust and independent of the identity of the rater (teacher or parent), exhibiting cross-group structural equivalence.

## 5. Discussion

Although the Learning and Development Guide for Children Aged 3–6 in China (2012) has pointed out the importance of children’s ATL, there are few instruments to effectively measure children’s ATL. The developed ATL scale in this study can be a reliable and effective instrument that is easy for teachers and parents to report. It identifies the reliability and effectiveness of children’s ATL factor structure. In particular, the study points out the need to develop instruments that are easily accessible to teachers and other practitioners in the daily educational process, because some instruments are time-consuming and restrictive in daily use. Therefore, the new scale of children’s learning products is not only an easy-to-use instrument in a short time in preschool classes but also a reliable and effective instrument for long-term observation and tracking.

This study developed a new instrument for teachers and parents to effectively assess the development level of children’s ATL. Prior research has shown that children with positive ATL exhibit better learning performance, and positive ATL have been underlined in policy documents and early education standards in multiple nations (Kagan, 1995; Yan and Wei, 2013; Peng, 2020). Previous studies have also suggested that if we use more factors to assess children’s ATL, the connotation of ATL becomes too broad to grasp (Chen and McNamee, 2011; McDermott et al., 2011; Huang et al., 2019). It is more important to unravel the core elements of ATL. Based on the policy requirements of China’s preschool education, our research focuses on the core elements of children’s ATL to establish its four-factor structure (i.e., creativity, learning strategy, competence motivation, and attention/persistence), which can be used to effectively assess children’s ATL and provide practical instruments for preschool teachers and parents to effectively train children’s ATL.

Previous studies rarely use “creativity” in the factor structure for ATL, assuming that creativity is irrelevant to children’s learning. However, creativity is the driving force for children to learn, which is one of children’s more important potentials. Children can use creative imagination to expand their knowledge, carry out new learning, generate new ideas, discover and create new things, and express themselves in unique ways (Yan-Wei, 2013). In this study, the measure of ATL and the factor of “creativity” highlight the way that children show their innate ability and describe the novelty in children’s thinking and use of materials. For example, through imagination, children can show their uniqueness and particularities through various activities, give full play to their imagination, and show confidence and positivity in the face of new events.

In addition, previous studies mostly measured children’s ATL from the single perspective of teachers and professionals, while less research adopted the perspective of parents (Barbu et al., 2015). However, it is necessary to develop instruments that teachers and other practitioners can easily use in the daily educational environment because many of the existing measurement instruments are too time-consuming and thus unsuitable for daily practice (Diamond et al., 2013). The scale developed in this study is based on the simultaneous use of two groups, namely teachers and parents, to assess children’s ATL. The scale contains four identical factors across the two reporting groups. The number of items is also appropriate and is not affected by rater identity. In sum, this paper contributes a new instrument for convenient but effective assessment of children’s ATL. ATL can enhance or reduce children’s learning outcomes (Hyson, 2008). Positive ATL will promote children’s academic achievements, while negative ATL will hinder children’s acquisition of knowledge or skills.

Children with learning difficulties need early detection and intervention. In this sense, the developed scale of approaches to learning provide us with a new screening tool. When encountering learning difficulties, children with positive ATL are more likely to show initiative, persistence, flexibility, and creativity, which can help children reduce the incidence of learning errors. ATL assessment can identify children with potential learning difficulties, to intervene in children with learning difficulties as soon as possible. In addition, even if children make learning mistakes, children with positive ATL can easier to learn from mistakes. Learning difficulties need early diagnosis and prevention. ATL has become a screening device for children with learning difficulties. Early detection, diagnosis, and intervention of children with learning difficulties will help promote the development of children’s academic performance and social–emotional ability (Chen and Huang, 2018).

## 6. Limitations and future research

The research sample in this study only represented a single province of China, which could limit the sample representativeness of this study to a certain extent, so caution should be used when applying the scale across regions. Future research can collect samples from multiple regions to confirm the structure of the developed scale. Although the sample size of this study seems adequate relative to relevant works in the ATL literature, future research can collect more samples to provide stronger evidence for the psychometric properties of the new ATL scale in our research. After the statistical analysis of preschoolers’ approaches to the learning scale, the inappropriate items were deleted according to the standards suggested in the literature, but these items may remain in other samples in future research. In this study, we completed the preparation of the scale and developed a reasonable structure and scientific content. Future research should explore the individual differences in sex, class, and parents’ economic and cultural levels to advance the understanding of factors affecting the development of children’s ATL and advance more comprehensive strategies to cultivate children’s ATL.

## 7. Conclusion

The approaches to learning of preschoolers are crucial for their development and future success. Effectively assessing preschoolers’ approaches to learning is thus important for parents and teachers to identify potential learning problems of preschoolers and implement timely intervention. However, there is a lack of a valid assessment instrument that can adapt to the Chinese context and be used by both teachers and parents. In this regard, this study develops an easy-to-use assessment instrument for preschool teachers and parents to measure approaches to learning in China, and for scholars who are interested in the current status and development of Chinese children’s approaches to learning. Both the results of EFA and CFA supported the 4-factor structure (creativity, learning strategy, competence motivation, and attention/persistence). Using social skills and executive function to examine the predictive validity of the approaches to learning scale, we find that all dimensions of the scale can positively predict children’s social skills and negatively predict children’s executive function, which is consistent with the findings of previous studies (McDermott et al., 2012; Wu et al., 2016). Multi-group CFA further showed that the measurement model was robust and had cross-evaluator stability. Particularly, creativity was found as a new dimension of approaches to learning in the Chinese context, contributing to the literature stream of approaches to learning with a context-specific factor and possibly spurring more researchers to examine the cross-national or even cross-cultural differences in the future. Overall, this new instrument can be used as an effective instrument for educators to assess children’s approaches to learning and for future researchers to investigate various factors influencing the development of children’s approaches to learning in the field of early childhood.

## Data availability statement

The original contributions presented in the study are included in the article/Supplementary material, further inquiries can be directed to the corresponding author.

## Ethics statement

This research was conducted in accordance with the ethical guidelines of the American Psychological Association and was approved by the Institutional Review Board (IRB) of Ningbo University.

## Author contributions

LF contributed to conception and design of the study, performed the statistical analysis, and wrote the first draft of the manuscript. JW provided substantial support for data collection and statistical analysis. All authors contributed to the manuscript revision and approved the submitted version.

## Funding

This study was supported by grants from the National Social Science Fund of China “Multilevel Modeling on the Influence of Parent Involvement on Children’s Approaches to Learning” (Grant No. BHA190151).

## Conflict of interest

The author declares that the research was conducted in the absence of any commercial or financial relationships that could be construed as a potential conflict of interest.

## Publisher’s note

All claims expressed in this article are solely those of the authors and do not necessarily represent those of their affiliated organizations, or those of the publisher, the editors and the reviewers. Any product that may be evaluated in this article, or claim that may be made by its manufacturer, is not guaranteed or endorsed by the publisher.

## References

[ref2] Barbu O. C. Marx R. W. Yaden D. B. Levine-Donnerstein D. (2015). Measuring approaches to learning in preschoolers: validating the structure of an instrument for teachers and parents. Education 44, 698–714. doi: 10.1080/03004279.2015.1024273

[ref3] Bodovski K. Fa Rkas G. (2008). "Concerted cultivation" and unequal achievement in elementary school. Soc. Sci. Res. 37, 903–919. doi: 10.1016/j.ssresearch.2008.02.007

[ref4] Bulotsky-Shearer R. J. Fernandez V. Dominguez X. Rouse H. L. (2011). Behavior problems in learning activities and social interactions in head start classrooms and early reading, mathematics, and approaches to learning. Sch. Psychol. Rev. 40, 39–56. doi: 10.1080/02796015.2011.12087727

[ref1002] Carmines E. G. Zeller R. A. (1979). Reliability and validity assessment. Sage publications.

[ref6] Chen Y. Huang S. (2018). Approaches to learning: a new perspective of preventing Children’s learning difficulties. Chin. J. Spec. Educ. 12, 50–68.

[ref7] Chen J. Q. Mcnamee G. D. (2011). Positive approaches to learning in thecontext of preschool classroom activities. Early Childhood Educ. J. 39, 71–78. doi: 10.1007/s10643-010-0441-x

[ref8] Crosby E. G. French J. L. (2010). Psychometric data for teacher judgments regarding the learning behaviors of primary grade children. Psychol. Sch. 39, 235–244. doi: 10.1002/pits.10034

[ref9] Diamond K. E. Justice L. M. Siegler R. S. Snyder P. A. (2013). Synthesis of ies research on early intervention and early childhood education [executive summary]. Nat. Center Spec. Educ. Res. 139:90. doi: 10.2139/ssrn.535115

[ref10] Fantuzzo J. Bulotsky-Shearer R. McDermott P. A. McWayne C. Frye D. Perlman S. (2007). Investigation of dimensions of social-emotional classroom behavior and school readiness for low-income urban preschool children. Sch. Psychol. Rev. 36, 44–62. doi: 10.1080/02796015.2007.12087951

[ref11] Feng L. (2020). The mediation effect of home learning environment between family socioeconomic status and preschool children’s approaches to learning. Stud. Early Child. Educ. 304, 62–72. doi: 10.13861/j.cnki.sece.2020.04.007

[ref12] Fornell C. Larcker D. F. (1981). Evaluating structural equation models with unobservable variables and measurement error. J. Mark. Res. 18, 39–50. doi: 10.1177/002224378101800104, PMID: 33691717

[ref13] Gresham F. M. Elliott S. N. Vance M. J. Cook C. R. (2011). Comparability of the social skills rating system to the social skills improvement system: content and psychometric comparisons across elementary and secondary age levels. Sch. Psychol. Q. 26, 27–44. doi: 10.1037/a0022662

[ref14] Hu L. T. Bentler P. M. (1999). Cut points for fit indices in covariance structure analysis: conventional criteria versus new alternatives. Struct. Equ. Model. Multidiscip. J. 1, 130–149.

[ref15] Hu B. Y. Teo T. Nie Y. Wu Z. (2017). Classroom quality and Chinese preschool children's approaches to learning. Learn. Individ. Differ. 54, 51–59. doi: 10.1016/j.lindif.2017.01.007, PMID: 36851900

[ref16] Huang S. Huo L. Fang Y. (2019). Progress of the research on nature and structure of approaches to learning in foreign countries. Int. Comp. Educ. 351, 106–112.

[ref17] Hyson M. (2008). Enthusiastic and engaged learners: ATL in the early childhood classroom. Newark: Educational Science Press.

[ref19] Kagan S. L.. (1995). Reconsidering Children’s Early Development and Learning: Toward Common Views and Vocabulary. Washington, DC: US Government Printing Office, Superintendent of Documents, Mail Stop, SSOP.

[ref20] Kaiser H. F. Rice J. (1974). Little jiffy, mark iv. Educ. Psychol. Meas. 34, 111–117. doi: 10.1177/001316447403400115

[ref21] Li-Grining C. P. Votruba-Drzal E. Maldonado-Carreño C. Haas K. (2010). Children's early approaches to learning and academic trajectories through fifth grade. Dev. Psychol. 46, 1062–1077. doi: 10.1037/a0020066, PMID: 20822223

[ref22] MacKenzie S. B. Podsakoff P. M. Podsakoff N. P. (2011). Construct measurement and validation procedures in MIS and behavioral research: integrating new and existing techniques. MIS Q. 35:293. doi: 10.2307/23044045

[ref24] McClelland M. M. Acock A. C. Morrison F. J. (2006). The impact of kindergarten learning-related skills on academic trajectories at the end of elementary school. Early Child. Res. Q. 21, 471–490. doi: 10.1016/j.ecresq.2006.09.003

[ref25] McClelland M. M. Morrison F. J. Holmes D. L. (2000). Children at risk for early academic problems: the role of learning-r-elated skills. Early Child. Res. Q. 15, 307–329. doi: 10.1016/S0885-2006(00)00069-7

[ref26] McDermott P. A. Beitman B. S. (1984). Standardization of a scale for the study of children’s learning styles: structure, stability, and criterion validity. Psychol. Sch. 21, 5–14. doi: 10.1002/1520-6807(198401)21:1<5::AID-PITS2310210102>3.0.CO;2-B

[ref27] McDermott P. A. Fantuzzo J. W. Warley H. P. Waterman C. Angelo L. E. Gadsden V. L.. (2011). Multidimensionality of teachers' graded responses for preschoolers' stylistic learning behavior: the learning-to-learn scales. Educ. Psychol. Meas. 71, 148–169. doi: 10.1177/0013164410387351

[ref28] McDermott P. A. Leigh N. M. Perry M. A. (2002). Development and validation of the preschool learning behaviors scale. Psychol. Sch. 39, 353–365. doi: 10.1002/pits.10036, PMID: 36384255

[ref29] McDermott P. A. Mordell M. Stoltzfus J. C. (2001). The organization of student performance in American schools: discipline, motivation, verbal learning, nonverbal learning. J. Educ. Psychol. 93, 65–76. doi: 10.1037/0022-0663.93.1.65

[ref30] McDermott P. A. Rikoon S. H. Fantuzzo J. W. (2014). Tracing children’s approaches to learning through head start, kindergarten, and first grade: different pathways to different outcomes. J. Educ. Psychol. 106, 200–213. doi: 10.1037/a0033547

[ref31] McDermott P. A. Rikoon S. H. Fantuzzo J. W. (2016). Transition and protective agency of early childhood learning behaviors as portents of later school attendance and adjustment. J. Sch. Psychol. 54, 59–75. doi: 10.1016/j.jsp.2015.10.003, PMID: 26790703

[ref32] McDermott P. A. Rikoon S. H. Waterman C. Fantuzzo J. W. (2012). The preschool learning behaviors scale: dimensionality and external validityin head start. Sch. Psychol. Rev. 41, 66–81. doi: 10.1080/02796015.2012.12087376

[ref34] Ministry of Education of the People’s Republic of China. (2012). *Guidelines for 3–6 Years Old Children’s Learning and Development*. Available at: http://www.moe.edu.cn/publicfiles/business/htmlfiles/moe/s3327/201210/xxgk_143254.html

[ref35] Peng D. (2020). The essence，factor structure and learning effects of approaches to learning in early childhood. Stud. Preschool Educ. 303, 57–65. doi: 10.13861/j.cnki.sece.2020.03.005

[ref36] Price L. (2004). Individual differences in learning: cognitive control, cognitive style, and learning style. Educ. Psychol. 24, 681–698. doi: 10.1080/0144341042000262971, PMID: 36682508

[ref37] Qian Z. Ding P. (2010). The development of diagnostic scale on the maturity of the children entering school. Stud. Preschool Educ. 182, 41–45. doi: 10.13861/j.cnki.sece.2010.02.001

[ref38] Razza R. A. Martin A. Brooks-Gunn J. (2015). Are approaches to learning in kindergarten associated with academic and social competence similarly? Child Youth Care Forum 44:757. doi: 10.1007/s10566-015-9307-0, PMID: 26877624 PMC4749025

[ref39] Rikoon S. H. Mcdermott P. A. Fantuzzo J. W. (2012). Approaches to learning among head start alumni: structure and validity of the learning behaviors scale. Sch. Psychol. Rev. 41, 272–294. doi: 10.1080/02796015.2012.12087509

[ref41] Scott-Little C. Kagan S. L. Frelow V. S. (2006). Conceptualization of readiness and the content of early learning standards: the intersection of policy and research? Early Child. Res. Q. 21, 153–173. doi: 10.1016/j.ecresq.2006.04.003

[ref42] Serife A. (2008). A conceptual analysis on the approaches to learning. Educ. Sci. 8, 707–720.

[ref43] Sparkman R. M. Hair J. F. Anderson R. E. Tatham R. L. Grablowsky B. J. (1979). Multivariate data analysis with readings. J. Mark. Res. 16:437. doi: 10.2307/2983017

[ref44] Spicer J. (2005). Making sense of multivariate data analysis: annals of pharmacotherapy. Thousand Oaks, CA: Sage.

[ref45] Strauss M. E. Smith G. T. (2009). Construct validity: advances in theory and methodology. Annu. Rev. Clin. Psychol. 5, 1–25. doi: 10.1146/annurev.clinpsy.032408.153639, PMID: 19086835 PMC2739261

[ref46] Sun X. Zhou Z. (2005). Exploratory factor analysis and its main problemsin application. Psychol. Sci. 28, 1440–1442. doi: 10.16719/j.cnki.1671-6981.2005.06.039

[ref47] Thorell L. B. Nyberg L. (2008). The childhood executive functioning inventory (chexi): a new rating instrument for parents and teachers. Dev. Neuropsychol. 33, 536–552. doi: 10.1080/87565640802101516, PMID: 18568903

[ref48] Wang B. Feng X. Xiao S. Cang C. (2010). Family SES, approach to learning and school readiness. Stud. Preschool Educ. 4, 3–9. doi: 10.13861/j.cnki.sece.2010.04.015

[ref49] Wei W. Xie Q. B. Zhu J. J. He W. Li Y. (2018). The psychometric characteristics of childhood executive functioning inventory among Chinese preschoolers. Chin. J. Clin. Psych. 26, 26–29. doi: 10.16128/j.cnki.1005-3611.2018.01.006

[ref50] Wu Z. Hu B. Y. Fan X. (2016). Cross-cultural validity of preschool learning behavior scale in Chinese cultural context. J. Psychoeduc. Assess. 37, 125–130. doi: 10.1177/0734282916651538

[ref51] Yan C. Wei T. (2013). Interpretation of approach toward learning in guideto learning and development of 3-6 year old children. Early Child. Educ. 586, 1–5. doi: 10.16187/j.cnki.issn1001-4918.2013.03.004

[ref52] Yan-Wei L. I. (2013). The effects of home learning environment on children'searly academic and social skills. Psychol. Dev. Educ. 3, 268–274. doi: 10.16187/j.cnki.issn1001-4918.2013.03.004

[ref53] Ye D. Guo L. (2021). A study on preschool teachers’ understanding of approaches to learning. Early Child. Educ. 889, 27–32.

[ref54] Yue Y. Ren Y. (2021). The influence of family support on the approaches to learning of 5~6 years old children. Stud. Preschool Educ. 319, 5–16. doi: 10.13861/j.cnki.sece.2021.07.002

[ref1001] Zhao J. Wang X. (2018). Observing and Evaluating Scale of 3~6-year-old Children’s Approaches toward Learning. Early Child. Educ. 44–59. doi: 10.13861/j.cnki.sece.2018.06.005

[ref55] Ziv Y. (2013). Social information processing patterns, social skills, and schoolreadiness in preschool children. J. Exp. Child Psychol. 114, 306–320. doi: 10.1016/j.jecp.2012.08.009, PMID: 23046690 PMC3508340

