# Supplementary material for: Assessing preschoolers’ approaches to learning in the Chinese context: a scale for teacher-parent co-evaluation
Source: Front Psychol. 2023 Jun 2;14:1098506. doi: 10.3389/fpsyg.2023.1098506 (PMC10273840; doi:10.3389/fpsyg.2023.1098506)
Supplement: Supplementary file 1 [file Data_Sheet_1.pdf]

Supplementary Material

Supplementary Figures

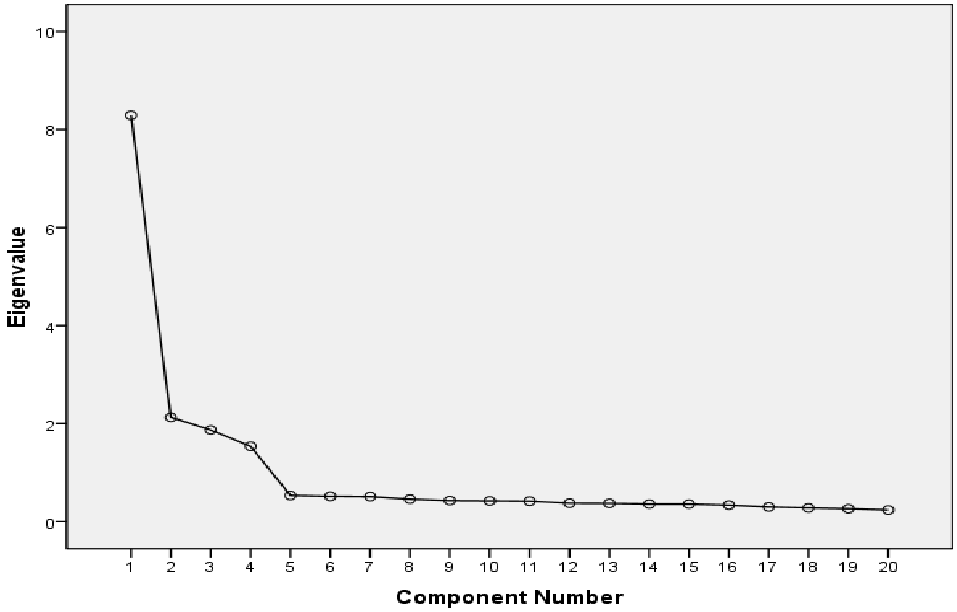

Figure A1. Scree plot of exploratory factor analysis (Teacher Version).

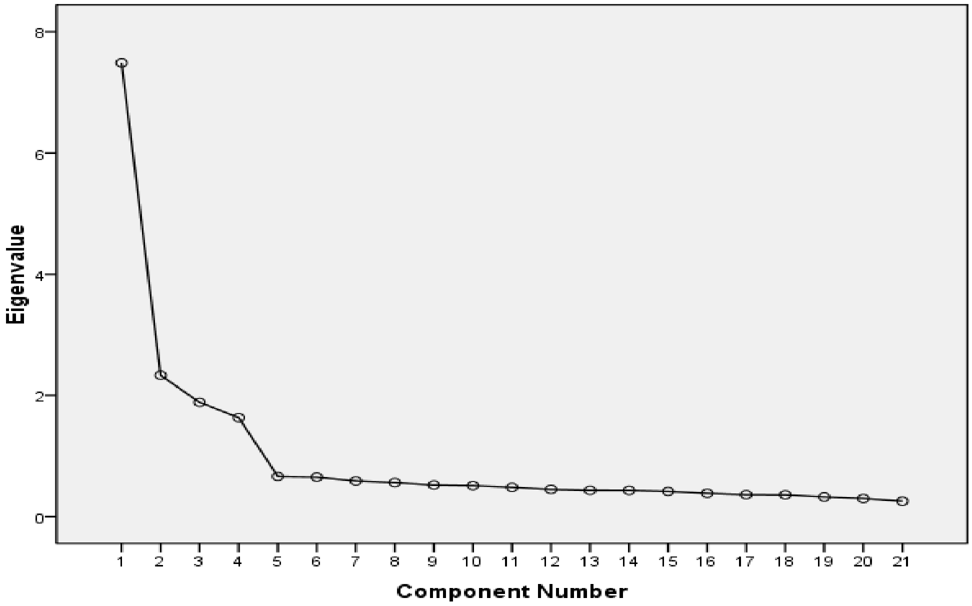

Figure A2. Scree plot of exploratory factor analysis (Parent Version).

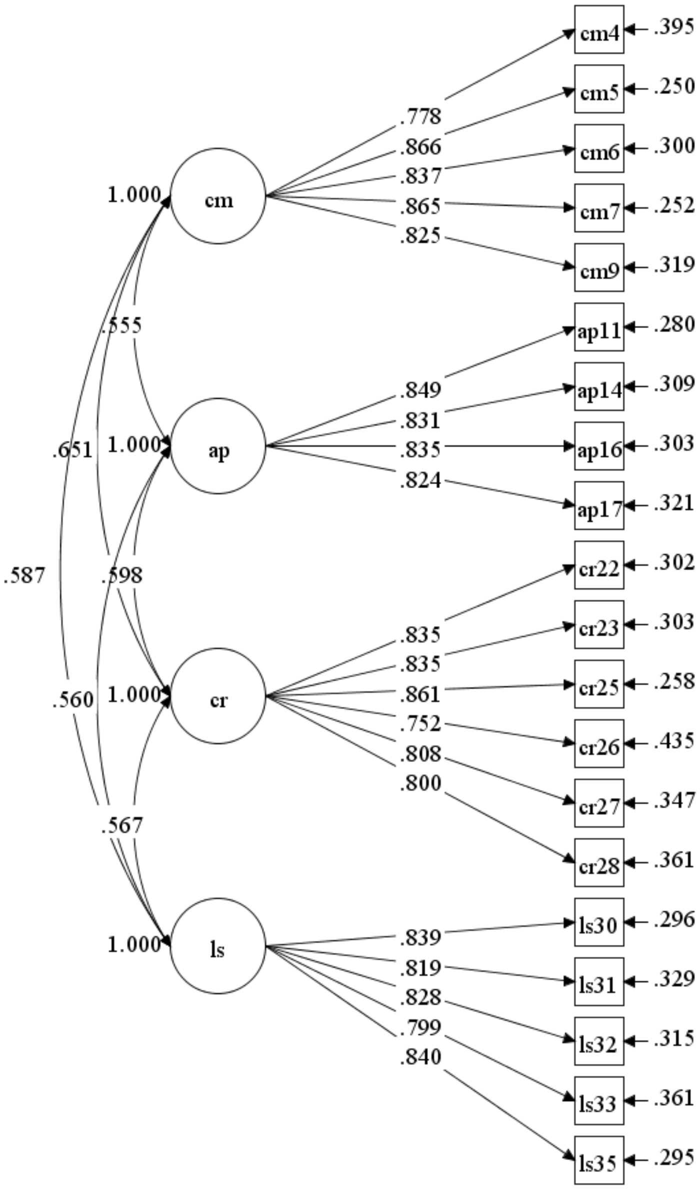

Figure A3. Results of multi-group CFA (Teacher Version).

Note: cm - competence motivation, ap - attention/persistence, cr - creativity, ls - learning strategy.

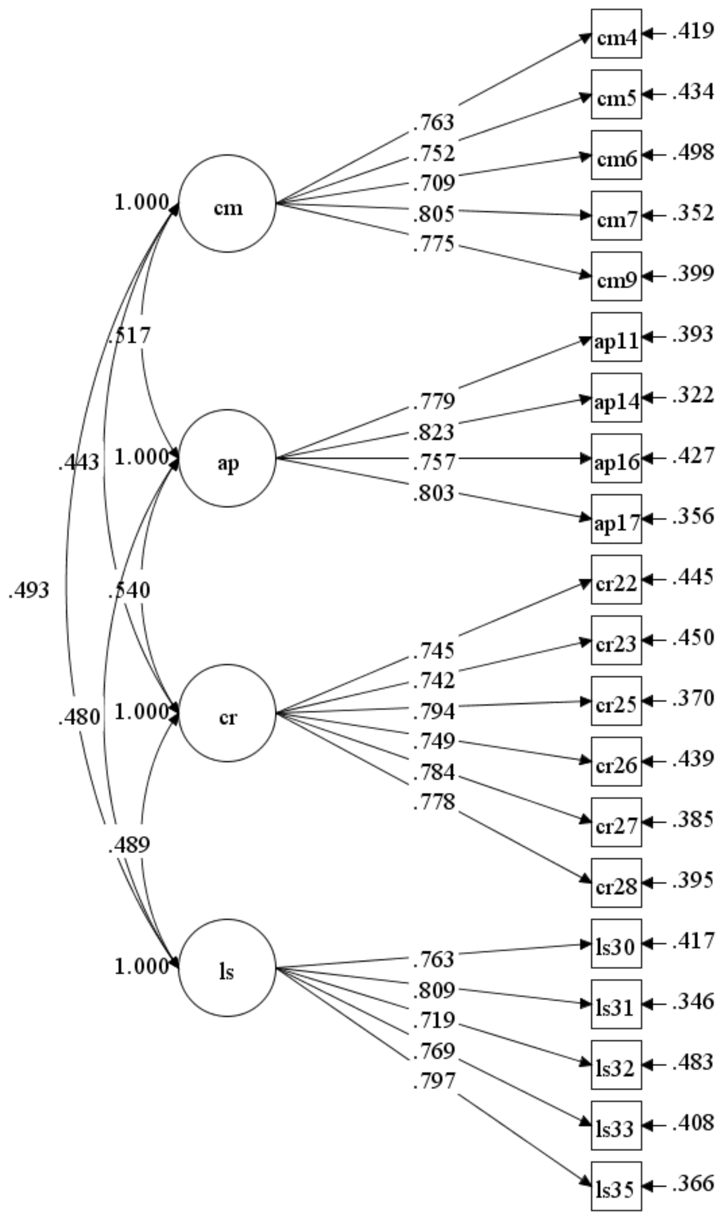

Figure A4. Results of multi-group CFA (Parent Version).

Note: cm - competence motivation, ap - attention/persistence, cr - creativity, ls - learning strategy.

Supplementary Tables

Table A1. Results of eigenvalues and explained variance proportion (Teacher Version).

	Initial Eigenvalues			Rotation Sums of Squared Loadings		
Component	Total	% of Variance	Cumulative %	Total	% of Variance	Cumulative %
1	8.290	41.449	41.449	3.993	19.964	19.964
2	2.126	10.629	52.078	3.550	17.748	37.712
3	1.870	9.349	61.426	3.530	17.652	55.364
4	1.538	7.688	69.115	2.750	13.751	69.115

Table A2. Results of eigenvalues and explained variance proportion (Parent Version).

	Initial Eigenvalues			Rotation Sums of Squared Loadings		
Component	Total	% of Variance	Cumulative %	Total	% of Variance	Cumulative %
1	7.485	35.643	35.643	3.735	17.785	17.785
2	2.331	11.099	46.743	3.339	15.899	33.683
3	1.885	8.974	55.717	3.262	15.533	49.216
4	1.631	7.766	63.482	2.996	14.266	63.482

Table A3. Convergent validity of ATL scale.

Dimension	Items	AVE (Teacher Version)	AVE (Parent Version)
Competence Motivation	5	0.697	0.580
Attention/Persistence	4	0.695	0.626
Creativity	6	0.666	0.586
Learning Strategy	5	0.681	0.596
